# Knowledge, use and attitudes of healthcare professionals towards patient-reported outcome measures (PROMs) at a comprehensive cancer center

**DOI:** 10.1186/s12885-022-09269-x

**Published:** 2022-02-10

**Authors:** Cinzia Brunelli, Emanuela Zito, Sara Alfieri, Claudia Borreani, Anna Roli, Augusto Caraceni, Giovanni Apolone

**Affiliations:** 1 Palliative Care, Pain Therapy and Rehabilitation Unit, Fondazione IRCCS Istituto Nazionale dei Tumori, Milan, Italy; 2 Information and Communication Technology Unit, Fondazione IRCCS Istituto Nazionale dei Tumori, Milan, Italy; 3 Clinical Psychology Unit, Fondazione IRCCS Istituto Nazionale dei Tumori, Via Venezian 1, 20133 Milan, Italy; 4 Quality, Education and Data Protection Unit, Fondazione IRCCS Istituto Nazionale dei Tumori, Milan, Italy; 5 Scientific Directorate, Fondazione IRCCS Istituto Nazionale dei Tumori, Milan, Italy

**Keywords:** Oncology, PROMs, Patient-reported outcome, Quality of life

## Abstract

**Background:**

Despite evidence of the positive impact of routine assessment of patient-reported outcome measures (PROMs), their systematic collection is not widely implemented in cancer care.

**Aim:**

To assess the knowledge, use and attitudes of healthcare professionals (HCPs) towards PROMs and electronically collected PROMs (ePROMs) in clinical practice and research and to explore respondent-related factors associated with the above dimensions.

**Method:**

An ad hoc developed online survey was administered to all HCPs employed in clinical activity in an Italian comprehensive cancer center. The survey investigated which PROMs were known and used, as well as HCPs’ opinions on the advantages and drawbacks of routine PROM assessment, including electronic assessment (ePROM). Linear and logistic regression models were used for association analyses.

**Results:**

Five Hundred Eleven of nine hundred ninety-two invited HCPs (52%) provided analyzable responses. 68% were women, 46% were nurses and 42% physicians, and 52.5% had > 20 years seniority. The average number of PROMs known was six among 17 proposed. All proved to be under-used (< 28%) except unidimensional and multidimensional pain scales (77 and 36%). Respondents expressed an overall positive attitude towards PROMs, with strengths outweighing weaknesses (mean overall scores 3.6 and 2.9, respectively, on a 1–5 scale). 67% of respondents preferred electronic collection over paper and pencil. Profession was associated with knowledge and use (physicians reported knowing more PROMs than other professionals) and with a preference for electronic collection (nurses were less likely to prefer the electronic format than physicians). Senior HCPs were slightly more critical about both PROMs and electronic administration.

**Conclusions:**

This survey indicates an acceptable level of knowledge of common PROM tools but low usage in practice. Based on the generally positive attitude of HCPs, routine implementation of ePROMs can be promoted as long as adequate resources and training are provided.

**Trial registration:**

Not registered.

**Supplementary Information:**

The online version contains supplementary material available at 10.1186/s12885-022-09269-x.

## Introduction

Patient-reported outcome measures (PROMs) are standardized questionnaires completed by patients. Their purpose is to assess patients’ perception of a variety of health and well-being indicators that provide important information to healthcare professionals (HCPs) for patient care. PROMs have been advocated for use in routine cancer care for some time now [1] and there is evidence that they may improve symptom control, patient well-being, cost effectiveness, patient engagement and survival [2–8]. Routine use of PROMs is also considered a major indicator of integration between oncology and palliative care [9].

Reviews of the literature have shown that HCPs perceive PROMs as facilitating the identification and assessment of symptoms and of psychological, social and spiritual distress [10–12]. Also patients perceive PROMs as relevant, easy to use and useful for describing their health-related conditions [13, 14]. However, systematic PROM collection is not widely implemented in routine oncology practice for individual patient care [15–17]. Difficulty in changing established work practices, a lack of time, and fear of a negative impact on the patient-clinician relationship are the main causes of their limited use [13, 18, 19]. Electronic assessment of PROMs (ePROMs) has been identified as a potential means to overcome barriers to routine use [20, 21] and an opportunity to maintain and further develop a patient-centered approach to care in the era of big data [22].

Although the findings on these topics are progressively increasing, there is a lack of studies evaluating the knowledge and use of different PROMs, and there are still few reports on HCPs’ attitudes towards ePROMs. Within a wider project called *Patient Voices* [23] aimed at promoting the use of PROMs in routine cancer care at an Italian comprehensive cancer center, the present survey has the purpose of assessing the knowledge, use and attitudes of HCPs towards PROMs and ePROMs in clinical practice and research and of exploring respondent-related factors associated with the above dimensions.

## Methods

### Participants

All HCPs (physicians, nurses, psychologists, physiotherapists, radiotherapy and radiology technicians) employed in clinical work at the Fondazione IRCCS Istituto Nazionale dei Tumori of Milan (INT) in January 2019 (*N* = 992) were eligible for the study. INT is a comprehensive cancer center associated to the Organization of European Cancer Institutes, pursuing the prevention, early diagnosis and treatment of cancer. Eligible HCPs were invited to participate in an anonymous web survey sent through the SurveyMonkey® system. Survey reminders were sent to non-responders until a response rate of at least 50% was reached [24].

### Assessment

An ad hoc questionnaire was developed to investigate two main areas: 1) knowledge and use of PROMs; 2) attitudes of HCPs towards PROM and ePROM use in clinical practice. Data on sex, professional role, hospital department of affiliation, and seniority (years since degree/diploma) were also collected.

#### Knowledge and use of PROMs

A list of 17 tools for PROM collection commonly used in oncology to assess multiple dimensions of quality of life, psychological distress and physical symptoms was presented to the survey participants. The full list is reported in supplementary Table 1. For each tool respondents were asked to choose one of four possible options (1 = I don’t know the tool; 2 = I know the tool but have never used it; 3 = I have used the tool occasionally; 4 = I have used the tool frequently). Three further yes/no questions investigated the use of other tools not included in the list, the use of ad hoc developed (not validated) tools, and the use of self-translated (not validated) tools. One item investigated whether the respondent had used PROMs mainly in research, clinical practice or equally in both settings. Finally, two multiple choice questions asked which professional role among a list of five (physician, nurse, psychologist, social worker, volunteer) should present and propose the completion of a PROM to the patient and which of them should assist the patient in completing it.

#### Attitudes and opinions about PROMs

The survey continued with a list of 17 statements on PROM strengths (e.g. “PROMs can be useful for documenting the quality of care we offer to patients”) and weaknesses (e.g. “During the visit there is no time for adequate administration of PROMs”). One item investigated participants’ preferred collection mode (electronic vs paper & pencil) and eight further items addressed the strengths and weaknesses of electronic PROM collection (e.g. “ePROMs allow a graphic display of the symptom and quality of life trend of scores over time” or “ePROMs are difficult to implement due to the lack of familiarity with electronic devices in some groups of patients”). Responses were collected through five-step Likert scales, from 1 = totally disagree to 5 = totally agree.

### Statistical analysis

When planning the survey we calculated that a sample size of 450 respondents would allow to estimate a two-sided 95% confidence interval (CI) for the mean scores with a precision (half CI width) of 9.2% of its standard deviation [25]. In case of a dichotomous answer to questionnaire items (yes/no) and in the conservative hypothesis of 50% positive answers, the precision would be 4.6% [25].

A data quality check was performed to identify – and eliminate from the analysis – careless or inattentive responding, as this is a source of measurement error that can obscure meaningful results [26]. The following indicators were calculated for each respondent:

- survey completion time less than 4 min
- identical answers to all items on PROM tool knowledge and use
- identical answers to all items about the strengths and weaknesses of PROMs
- identical answers to all items about the strengths and weaknesses of ePROMs
- a diagonal response pattern in at least one block of items (e.g. 12,341,234)

Respondents scoring positive on at least four of the above five indicators were removed from the analysis. Respondent versus non-responder characteristics were compared using the χ^2^ test for independence on aggregate data from the list of invited HCPs.

Average scores and their 95% CIs were calculated for single strength and weakness items of both PROMs and ePROMs. In order to reduce the dimensionality of the set of items regarding knowledge, use and attitudes towards PROMs and ePROMs we calculated the following summary scores with the corresponding Cronbach alpha (α), when relevant: number of PROMs known, number of PROMs used frequently, overall average score of PROM strength items (α = 0.93), overall average score of PROM weakness items (α = 0.85), overall average score of ePROM strength items (α = 0.87), and overall average score of ePROM weakness items (α = 0.63). Multivariate linear regression models were applied to determine if sex, profession (physician, nurse, other), seniority (years since degree/diploma) and hospital department (surgery, medical oncology & hematology, critical & supportive care, other) (independent variables) were associated with each of the six indicators mentioned above (dependent variables). Two separate multivariate logistic regression models were applied to assess the association of the same independent variables as above with use of PROMs in clinical practice and with preference for either a paper or electronic format. Due to subgroup sample size constraints, only first-order interactions among independent variables were examined in the regression models.

In evaluating regression models, statistical significance was deemed for *p* < 0.01 and 99% confidence intervals. Statistical analyses were performed using the Stata 16 software (StataCorp. 2019. Stata Statistical Software: release 16. College Station, TX: StataCorp LLC.).

The *Patient Voices* project, including the present survey, was approved by INT’s institutional review board (INT 167/18).

## Results

Of the 992 HCPs invited to the survey, 552 opened the link and viewed the questionnaire; 523 filled out the first two screens of the survey and were defined as respondents (response rate 52.3%). Data of 12 respondents were eliminated during the quality check described in the Methods section, leaving a final set of 511 participants. Among them, 475 completed the survey till the end (completion rate 47.9%). Respondents and non-responders were comparable with regard to profession (*p* = 0.28) and department of affiliation (*p* = 0.44), while respondents were slightly more frequently female (68% vs 62%, *p* = 0.048).

Table 1 shows the characteristics of the 511 participants: the majority were women (68.1%), the professions were mainly nurses (45.6%) and physicians (42.3%), working either in surgery (28.8%) or medical oncology and hematology (24.7%)departments; a small majority (52.5%) had > 20 years’ seniority.

**Table 1:** Characteristics of respondents (*N* = 511)

	N	%
**Sex**		
Female	348	68.1
Male	163	31.9
**Department of affiliation**		
Surgery	147	28.8
Critical & supportive care ^a^	110	21.5
Medical oncology & hematology	126	24.7
Other^b^	128	25.0
**Profession**		
Physician	216	42.3
Nurse	233	45.6
Psychologist	13	2.5
Physiotherapist	14	2.7
Radiotherapist or radiology technician	27	5.3
Other^c^	8	1.6
**Seniority (years since degree/diploma)**		
≤ 10	129	25.2
10–20	113	22.1
≥ 20	268	52.5
Missing	1	0.2

^a^ Palliative Care and Pain Therapy & Rehabilitation, Supportive Care, Clinical Psychology, Intensive Care, Pneumology, Parenteral Nutrition, Cardiology

^b^ Diagnostic Imaging and Radiotherapy, Medical Directorate

^c^ Social workers and dieticians

### Knowledge and use

**Fig. 1:**
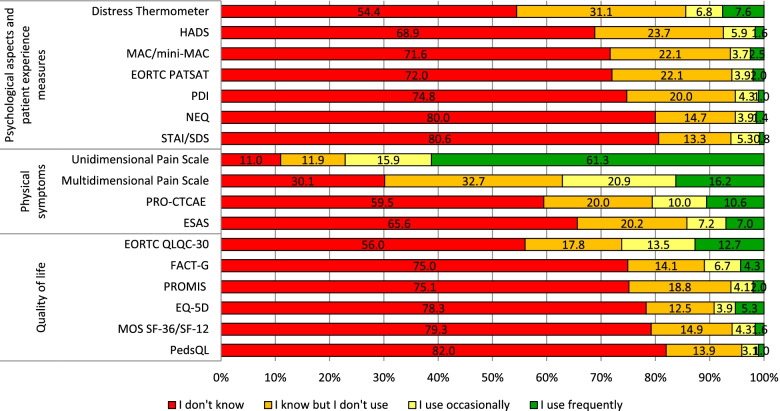
Percentage of known and used PROMs. Note: The full list of references and acronyms for the PROMs in Fig. 1 is included in Supplementary Table 1

Figure 1 shows data on the knowledge and use of 17 common PROM tools sorted by purpose (psychological aspects and patient experience measures, physical symptoms, quality of life). Unidimensional pain scales were the most frequently known (80.9%) and used tools (61.3%), followed by multidimensional pain scales, known by 69.9% of respondents and frequently used by 16.2%. On average, respondents reported knowing 5.8 tools (range 0–17) but having used at least occasionally only 2.6 (range 0–15), frequent use being even more limited (1.4 tools on average). A number of self-report questionnaires including PedsQL, STAI (or SDS), MOS SF-36 (or SF-12) and EQ-5D were reported to be little known by HCPs (“Do not know” > 75%). In general, physical symptom scales were more frequently known and used than quality of life, psychological aspects and patient experience measures.

Sixty-five respondents (13%) reported having used other PROMs not included in the list. PROMs reported at least twice were DN4, Breast-Q, IPSS, MMPI and Morse S (see Supplementary Table 1 for acronyms and references).

**Fig. 2:**
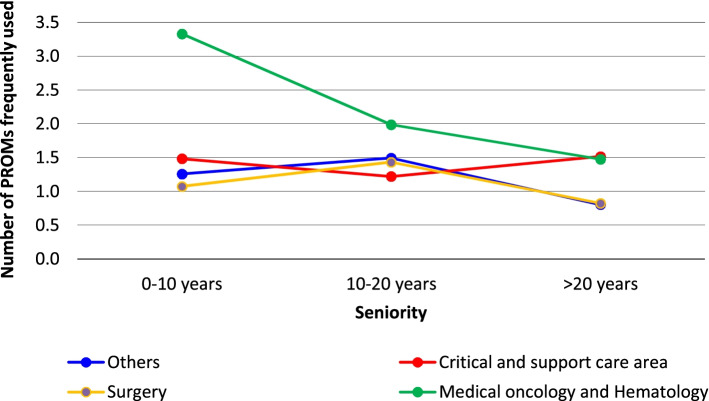
Regression model estimates of mean number of frequently used PROMs by hospital department and seniority

The multiple linear regression results indicated that profession and department were significantly associated with the number of PROMs known (Table 2, first column): nurses and “other professions” reported knowing 2.9 and 2.7 fewer tools, respectively, than physicians, while HCPs working in medical oncology and hematology departments reported knowing 1.9 more tools than those working in surgical departments. The number of PROMs used frequently (Table 2, second column) was the only regression model in which one interaction proved significant (between seniority and department of affiliation p), while none of the factors not included in the interaction emerged as significant. To facilitate model interpretation, we report in Fig. 2 the number of PROMs frequently used by various combinations of seniority and department as estimated by the model. While HCPs in medical oncology and hematology departments consistently reported a greater use of tools than other HCPs, the difference with other departments was much more pronounced for younger HCPs.

**Table 2:** Results of multiple linear and logistic regression models to explore respondent characteristics associated with knowledge, use and attitudes towards PROMs and ePROMs

	Number of PROMs known	Number of PROMs used	Use of PROMs in clinical practice	Overall PROM strengths score	Overall PROM weaknesses score	Preference for use of ePROMs	Overall ePROM strengths score	Overall ePROM weaknesses score
	β^a^ (99% CI)	β^a^ (99% CI)	OR^b^ (99% CI)	β^a^ (99% CI)	β^a^ (99% CI)	OR^a^ (99% CI)	β^a^ (99% CI)	β^a^ (99% CI)
**Sex**								
Female (ref.)	–	–	–	–	–	–	–	–
Male	−0.4	−0.03	1.1	0.0	- 0.1	1.6	− 0.1	**−0.2**
	−1.5 to 0.8	−0.4 to 0.3	0.6–2.0	− 0.2 to 0.3	− 0.3 to 0.1	0.8–2.9	−0.4 to 0.1	**− 0.5 to − 0.02**
**Seniority (years from degree)**								
0–10 (ref.)	–	–	–	–	–	–	–	–
10–20	0.1	0.4	0.9	−0.0	0.1	1.3	0.02	0.1
	−1.4 to 1.6	− 0.6 to 1.3	0.4–1.8	− 0.3 to 0.3	− 0.2 to 0.3	0.6–3.0	− 0.3 to 0.3	− 0.2 to 0.4
> 20	− 0.6	− 0.2	0.7	− 0.1	**0.3**	0.7	−0.03	**0.3**
	−1.8 to 0.6	− 1.0 to 0.5	0.4–1.3	− 0.4 to 0.1	**0.1 to 0.4**	0.4–1.3	−0.3 to 0.2	**0.1 to 0.6**
**Profession**								
Physician (ref.)	–	–	–	–	–	–	–	–
Nurse	**−3.0**	−0.2	0.9	−0.1	**− 0.2**	**0.5**	**− 0.3**	− 0.2
	**- 4.1 to − 1.8**	− 0.6 to 0.1	0.5–1.6	− 0.4 to 0.1	**− 0.4 to − 0.02**	**0.3 to 0.98**	**− 0.5 to − 0.1**	− 0.4 to 0.01
Other	**−2.7**	−0.5	0.5	0.2	−0.2	0.8	−0.0	−0.2
	**−4.5 to − 1.0**	−1.1 to 0.1	0.2–1.4	− 0.2 to 0.5	− 0.5 to 0.1	0.3 to 1.9	−0.4 to 0.3	− 0.5 to 0.2
**Department**								
Surgery (ref.)	–	–	–	–	–	–	–	–
Critical & supportive care	0.4	0.4	**2.0**	0.0	− 0.0	0.8	0.0	− 0.1
	− 1.0 to 1.9	−0.6 to 1.4	**1.0–4.1**	− 0.3 to 0.3	− 0.2 to 0.2	0.4–1.6	−0.3 to 0.3	− 0.4 to 0.2
Med. oncology and hematol.	**1.9**	**2.3**	1.2	0.1	−0.1	0.8	0.0	0.0
	**0.5 to 3.2**	**1.4 to 3.1**	0.6–2.3	− 0.2 to 0.4	−0.3 to 0.1	0.4–1.6	− 0.3 to 0.3	−0.3 to 0.3
Other (ref.)	−0.5	0.2	0.8	0.0	−0.1	1.0	0.0	−0.2
	−1.9 to 0.9	−0.7 to 1.1	0.4–1.8	− 0.3 to 0.3	− 0.3 to 0.1	0.5 to 2.2	− 0.3 to 0.3	−0.5 to 0.1
**Department by seniority Interaction**								
Critical & supportive care by seniority 10–20	–	−0.6	–	–	–	–	–	–
		−2.0 to 0.8						
Critical & supportive care by seniority > 20	–	0.3	–	–	–	–	–	–
		−0.9 to 1.4						
Med. oncology and hematol. by seniority 10–20	–	**−1.7**	–	–	–	–	–	–
		**−3.0 to − 0.35**						
Med. oncology and hematol.by seniority > 20	–	**−1.6**	–	–	–	–	–	–
		**−2.7 to − 0.53**						
Other department by seniority 10–20	–	−0.1	–	–	–	–	–	–
		−1.5 to 1.2						
Other department by seniority > 20	–	−0.2	–	–	–	–	–	–
		−1.3 to 0.9						
**Constant**	7.5 6.0 to 9.1	1.2 0.6 to 1.9	0.6 0.3–1.4	3.7 3.4–4.0	2.9 2.7 to 3.2	3.1 1.4 to 7.1	4.0 3.7 to 4.3	3.2 2.9 to 3.5
**N**	510	510	486	484	477	475	475	475

^a^ Multiple linear regression; ^b^ multiple logistic regression; *PROM* Patient-reported outcome measure, *ePROM* Electronic patient-reported outcome measure

Only 16 respondents (3.2%) declared having used PROMs developed ad hoc without any validation, and 15 (3%) reported having translated into Italian and used a questionnaire without any validation. Respondents opined that PROMs should be presented to patients preferably by nurses (64.4%) or physicians (61.4%) (data not reported in the table; more than one response was possible). Instead, it was mainly the nurse (63.6%) who was indicated as being responsible for assisting patients in completing the questionnaire. The figure in Supplementary Fig. 1 shows the detailed percentage distribution.

**Fig. 3:**
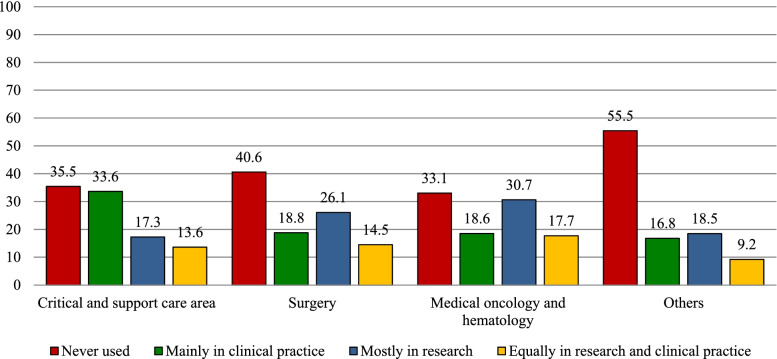
PROM use setting by hospital department

Two hundred and two respondents (41.5%) declared never having used any PROM tool, 111 (22.8%) having used them mainly in research and 106 (21.7%) in clinical practice, while 68 (14.0%) had used PROMs about equally in both settings. Figure 3 reports the PROM use setting by department, showing that the largest number of HCPs who had never used any PROM tool were in the department category “Other”. It also shows that PROMs were used mainly for research purposes by 30.1 and 25.6% of HCPs working in medical oncology/hematology and surgery departments, respectively, while the reported use for clinical practice purposes was around 19% for both; this ratio was reversed for HCPs working in critical & supportive care department, where PROM use for clinical practice was higher than that for research purposes (33.6% vs 17.6%). Overall, 174 respondents (35.7, 95% CI 31–40%) declared having used PROMs in clinical practice (mainly in clinical practice or equally in clinical practice and research). The multivariate logistic regression model results (Table 2, third column) indicated that use in clinical practice was not associated with any of the independent variables examined except for department, with HCPs in critical & supportive care department being more likely to use PROMs in clinical practice (OR = 2.0, 99% CI 1.0–4.1).

### Attitudes and opinions

Respondents showed a generally positive attitude towards the use of PROMs, with an overall average score of strengths outweighing weaknesses (3.6 vs 2.9, range 1–5) (Table 3). As regards weaknesses, items concerning the difficulty of administering PROMs due to a lack of resources (time, personnel) obtained higher average scores than items concerning a lack of PROM utility or reliability (Table 3). The multiple linear regression results indicated that none of the HCP characteristics examined were associated with the overall average score of PROM strengths (Table 2, fourth column), whereas seniority and profession were associated with weakness scores (Table 2, fifth column), albeit with quite low effect sizes. In particular, nurses had a less negative attitude than physicians (average weakness score 0.2 lower than physicians), while older HCPs expressed a more negative attitude than younger ones, with a 0.3-point higher average weakness score.

**Table 3:** Mean and 95% CI for PROM strengths and weaknesses: single-item and overall scores

	Mean	***95% CI***
**PROM strengths (*N*** **= 485)**		
PROMs can be useful to document the quality of care we offer our patients	3.7	*3.7–3.8*
PROMs help patients to express issues related to their health conditions	3.7	*3.6–3.8*
PROMs can provide information on problems I don’t investigate regularly	3.7	*3.6–3.8*
PROMs allow for wider and better patient involvement in the care process	3.6	*3.5–3.7*
PROMs facilitate patient relationships with the treating HCP team	3.5	*3.5–3.6*
PROMs repeatedly assessed over time are useful for clinical decision-making during the care process	3.5	*3.4–3.6*
PROMs allow more focused and efficient communication with the patient during the visit	3.5	*3.4–3.6*
Overall PROM strengths score	3.6	*3.5–3.7*
**PROM weaknesses (*N*** **= 477)**		
During the visit there is no time for adequate administration of PROMs	3.6	*3.5–3.7*
HCPs lack the resources to handle issues the patient may raise when completing PROMs	3.3	*3.2–3.4*
The administration of PROMs entails an additional workload for HCPs	3.3	*3.2–3.4*
PROMs are often filled in by caregivers	3.2	*3.2–3.3*
Patients have difficulties in understanding questionnaire response scales	3.1	*3.0–3.2*
PROMs fail to grasp the complexity of the patient’s experience	2.7	*2.6–2.8*
PROM scores are difficult to interpret	2.7	*2.6–2.8*
It is difficult to explain the use of PROMs to the patient	2.7	*2.6–2.8*
Completing PROMs is an excessive physical and psychological burden for patients	2.5	*2.5–2.6*
The data collected through PROMs are not reliable	2.4	*2.3–2.5*
PROMs do not add anything to the information HCPs already collect during the clinical contact	2.1	*2.0–2.2*
Overall PROM weaknesses score	2.9	*2.8–2.9*

*PROM* Patient-reported outcome measure, *HCP* Healthcare professional

Three hundred eighteen respondents (66.9, 95% CI 62.5–71.2) expressed a preference for the administration of PROMs in an electronic format. The multivariate logistic regression results (Table 2, sixth column) indicated that only profession was significantly associated with the preferred format: nurses were less likely to prefer ePROMs than physicians (OR = 0.5, 99% CI 0.3–0.98).

All items indicating ePROM strengths (Table 4) gained average scores close to 4 (range 1–5); in regard to weaknesses, respondents were mainly concerned with the potential lack of familiarity with electronic devices of some patients (average 3.4). Looking at the overall average scores, we found that ePROM strength scores outweighed weakness scores. The multiple linear regression results showed that nurses were slightly less positive than physicians about ePROMs (Table 2, seventh column), men were slightly less negative than women, and senior HCPs were slightly more critical than younger ones (Table 2, eighth column).

**Table 4:** Mean and 95% CI for ePROM strengths and weaknesses: single-item and overall scores (*N* = 475)

	Mean	95% CI
**ePROM strengths**		
ePROMs allow graphic display of symptom and quality of life trends over time	3.9	*3.8–4.0*
ePROMs facilitate scoring and score interpretation	3.9	*3.8–4.0*
ePROMs facilitate data sharing between different HCPs in the team	3.8	*3.7–3.9*
ePROMs allow remote patient follow-up	3.7	*3.6–3.8*
ePROMs facilitate the integration of PROM data with clinical data from other sources for research purposes	3.7	*3.6–3.8*
Overall score of ePROM strength items	3.8	*3.7–3.9*
**ePROM weaknesses**		
ePROMs are difficult to implement due to the lack of familiarity with electronic devices of some categories of patients	3.4	*3.3–3.5*
ePROMs will overload the EMR during the visit	3.1	*3.0–3.2*
ePROMs are difficult to implement due to data protection issues	2.8	*2.7–2.9*
Overall score of ePROM weakness items	3.1	*3.0–3.2*

*ePROM* Electronic patient-reported outcome measure, *HCP* Healthcare professional, *EMR* Electronic medical record

## Discussion

The results of this survey indicate that HCPs in a comprehensive cancer center, especially physicians, were familiar with a fair number of PROMs and expressed a generally positive attitude towards them. As reported in previous studies [10, 11, 16], the strengths of PROMs were considered to outweigh their weaknesses; however, frequent use was not common, which is also consistent with available evidence [10, 17, 27]. In particular, all tools but pain scales were reported to be used frequently only by a small percentage of respondents. It is worth noting that in Italy pain scales were introduced in hospital medical charts by a national law [28] in 2010 and their completion according to specific operating procedures has since been mandatory. This suggests that cultural and scientific developments may not be sufficient for successful implementation and that organizational and resource allocation interventions may be just as important [29].

In general, the fact that physical-symptom tools tend to be more frequently known and used than PROMs focused on quality-of-life or psychological aspects may be explained by the multiple barriers faced by oncologists in the assessment and management of mental health distress in patients with cancer [30].

Interestingly, the survey indicated that junior HCPs working in oncology/hematology departments reported using more tools than others, and that PROM use in research was more widespread in oncology/hematology and surgery departments than other areas. Likewise, senior HCPs identified more PROM weaknesses than younger colleagues. Such results are in agreement with the fact that junior HCPs are actively involved in data collection for research projects and also that they are taught about the value and use of PROMs in oncology care during their training. Consistent with these results is the more common use of PROMs in clinical practice in the critical & supportive care department, which includes palliative care, supportive care, clinical psychology as well as anesthesia and postoperative pain services. These are settings where patient care is mainly focused on physical and psychosocial symptoms, the assessment of which is strongly grounded on self-reporting.

Consistent with findings in the literature [10, 13, 19, 27], the perceived weaknesses of PROMs mainly concern the lack of resources and the additional workload for their administration, while concerns about their utility are less strong. Conversely, there is published evidence that routine PROM assessment allows more effective use of time and resources [11, 31]. The aspects related to concerns about the reliability of PROMs that have emerged in other studies [13, 19] were less common in the present survey results. PROM tools were originally developed for use in research, and perhaps their administration is still identified as a “research task” potentially interfering with clinical practice rather than an effective means of clinician-patient communication.

Around two-thirds of HCPs reported preferring the use of PROMs in an electronic rather than paper-and-pencil format, with nurses reporting lower ePROM strength scores. Since electronic PROM assessment was not a reality at INT when the survey was carried out, the expressed preferences are merely theoretical; however, respondents showed an overall positive attitude towards ePROMs, acknowledging their potential in making the process of data collection and use more efficient. Boyce et al. [10] reported that there may be concerns that technology diminishes the “human touch” in the care process and this may be explain why nurses were found to be slightly more critical towards ePROMs.

The present study has some limitations. Firstly, the survey was carried out in a single institution, a comprehensive cancer center with a tradition in health-related quality of life research. This may limit the generalization of the results, as they may not fully reflect oncology practice in other, less specialized and research-oriented settings. Secondly, the survey questionnaire was developed ad hoc; however, with the exception of the overall score of weaknesses of ePROMs, where Cronbach’s alpha value was at the limit of acceptability, all overall scores assessing attitudes had alpha values above 0.85.

## Conclusions

Although routine use of PROMs in clinical practice has shown a positive impact on patient care, management and even prognosis, their use in this comprehensive cancer center was still found to be less than optimal, confirming previous evidence. It is therefore important to plan specific efforts and appropriate strategies to achieve higher levels of implementation, which should be based on wide stakeholder involvement and application of national and international standardized procedures. Given the limited implementation of PROMs despite the relatively positive attitude of HCPs, the *Patient Voices* [23] project aims to fill this gap not only from a technological standpoint (through the development of an ePROM system integrated with the electronic medical record) but also from a cultural perspective, engaging both HCPs and patients in the choice and routine use of these tools for more effective patient care.

## Supplementary Information

**Additional file 1.**

## Data Availability

Data will be available from the corresponding authors [SA] on request.

## References

[1] Snyder CF, Aaronson NK (2009). Use of patient-reported outcomes in clinical practice. Lancet.

[2] Basch E (2017). Patient-reported outcomes—harnessing patients’ voices to improve clinical care. N Engl J Med.

[3] Basch E, Deal AM, Dueck AC, Scher HI, Kris MG, Hudis C (2017). Overall survival results of a trial assessing patient-reported outcomes for symptom monitoring during routine cancer treatment. JAMA.

[4] Denis F, Lethrosne C, Pourel N, Molinier O, Pointreau Y, Domont J, Bourgeois H, Senellart H, Trémolières P, Lizée T, Bennouna J, Urban T, El Khouri C, Charron A, Septans AL, Balavoine M, Landry S, Solal-CélignyP, Letellier C. Randomized trial comparing a web-mediated follow-up with routine surveillance in lung cancer patients. JNCI. 2017;109(9):djx029. 10.1093/jnci/djx029. 10.1093/jnci/djx029 28423407

[5] Lizée T, Basch E, Trémolières P, Voog E, Domont J, Peyraga G (2019). Cost-effectiveness of web-based patient-reported outcome surveillance in patients with lung cancer. J Thorac Oncol.

[6] Barbera L, Sutradhar R, Seow H, Earle CC, Howell D, Mittmann N (2020). Impact of standardized Edmonton symptom assessment system use on emergency department visits and hospitalization: results of a population-based retrospective matched cohort analysis. JCO Oncol Pract.

[7] Barbera L, Sutradhar R, Seow H, Mittmann N, Howell D, Earle CC (2020). The impact of routine Edmonton symptom assessment system (ESAS) use on overall survival in cancer patients: results of a population-based retrospective matched cohort analysis. Cancer Med.

[8] Barbera L, Sutradhar R, Earle CC, et al. The impact of routine Edmonton symptom assessment system use on receiving palliative care services: results of a population-based retrospective-matched cohort analysis. BMJ Support Palliat Care. 2020. 10.1136/bmjspcare-2020-002220. 10.1136/bmjspcare-2020-002220 32943469

[9] Kaasa S, Loge JH, Aapro M, Albreht T, Anderson R, Bruera E (2018). Integration of oncology and palliative care: a lancet oncology commission. Lancet Oncol.

[10] Boyce MB, Browne JP, Greenhalgh J (2014). The experiences of professionals with using information from patient-reported outcome measures to improve the quality of healthcare: a systematic review of qualitative research. BMJ Qual Saf.

[11] Howell D, Rosberger Z, Mayer C, Faria R, Hamel M, Snider A (2020). Personalized symptom management: a quality improvement collaborative for implementation of patient reported outcomes (PROs) in ‘real-world’oncology multisite practices. J Patient Rep Outcomes.

[12] Williams K, Sansoni J, Darcy M, Grootemaat P, Thompson C (2016). Patient-reported outcome measures. Literature review.

[13] Easpaig BNG, Tran Y, Bierbaum M, Arnolda G, Delaney GP, Liauw W (2020). What are the attitudes of health professionals regarding patient reported outcome measures (PROMs) in oncology practice? A mixed-method synthesis of the qualitative evidence. BMC Health Serv Res.

[14] Kotronoulas G, Kearney N, Maguire R, Harrow A, Di Domenico D, Croy S (2014). What is the value of the routine use of patient-reported outcome measures toward improvement of patient outcomes, processes of care, and health service outcomes in cancer care? A systematic review of controlled trials. J Clin Oncol.

[15] Bainbridge D, Seow H, Sussman J, Pond G, Martelli-Reid L, Herbert C (2011). Multidisciplinary health care professionals' perceptions of the use and utility of a symptom assessment system for oncology patients. J Oncol Pract.

[16] Chasen M, Bhargava R, Dalzell C, Pereira JL (2015). Attitudes of oncologists towards palliative care and the Edmonton symptom assessment system (ESAS) at an Ontario cancer center in Canada. Support Care Cancer.

[17] Pereira JL, Chasen MR, Molloy S, Amernic H, Brundage MD, Green E (2016). Cancer care Professionals' attitudes toward systematic standardized symptom assessment and the Edmonton symptom assessment system after large-scale population-based implementation in Ontario, Canada. J Pain Symptom Manag.

[18] Nguyen H, Butow P, Dhillon H, Sundaresan P. A review of the barriers to using patient-reported outcomes (PROs) and patient-reported outcome measures (PROMs) in routine cancer care. J Med Radiat Sci. 2020;68(2):186–95. 10.1002/jmrs.421 PMC8168064 32815314

[19] Roberts NA, Alexander K, Wyld D, Janda M (2019). What is needed by staff to implement PROMs into routine oncology care? A qualitative study with the multi-disciplinary team. European journal of cancer care.

[20] Basch E (2016). Missing patients’ symptoms in cancer care delivery—the importance of patient-reported outcomes. JAMA Oncol.

[21] LeBlanc TW, Abernethy AP (2017). Patient-reported outcomes in cancer care—hearing the patient voice at greater volume. Nat Rev Clin Oncol.

[22] Calvert M, Thwaites R, Kyte D, Devlin N (2015). Putting patient-reported outcomes on the ‘big data road map’. J R Soc Med.

[23] Brunelli C, Borreani C, Caraceni A, Roli A, Bellazzi M, Lombi L (2020). PATIENT VOICES, a project for the integration of the systematic assessment of patient reported outcomes and experiences within a comprehensive cancer center: a protocol for a mixed method feasibility study. Health Qual Life Outcomes.

[24] McPeake J, Bateson M, O’Neill A. Electronic surveys: how to maximise success. Nurse Res. 2014;24–26. 10.7748/nr2014.01.21.3.24.e1205. 10.7748/nr2014.01.21.3.24.e1205 24460562

[25] Machin D, Campbell M, Tan S, Tan S (1997). Sample size tables for clinical studies.

[26] Maniaci MR, Rogge RD (2014). Caring about carelessness: participant inattention and its effects on research. J Res Pers.

[27] Nguyen H, Butow P, Dhillon H, Morris L, Brown A, West K (2020). Using patient-reported outcomes (PROs) and patient-reported outcome measures (PROMs) in routine head and neck cancer care: what do health professionals perceive as barriers and facilitators?. J Med Imaging Radiat Oncol.

[28] NEGRI F. L’accesso alle cure palliative e alla terapia del dolore alla luce della nuova legge 15 marzo 2010 n. 38, in Sanità pubblica e privata. 2011;3:15–24.

[29] Schmidt T, Valuck T, Perkins B, Riposo J, Patel P, Westrich K (2021). Improving patient-reported measures in oncology: a payer call to action. J Managed Care Specialty Pharm.

[30] Granek L, Nakash O, Ariad S, Shapira S, Ben-David M (2018). Oncologists’ identification of mental health distress in cancer patients: strategies and barriers. Eur J Cancer Care.

[31] Rotenstein LS, Huckman RS, Wagle NW (2017). Making patients and doctors happier—the potential of patient-reported outcomes. N Engl J Med.

