# Supplementary material for: Knowledge, use and attitudes of healthcare professionals towards patient-reported outcome measures (PROMs) at a comprehensive cancer center
Source: BMC Cancer. 2022 Feb 10;22:161. doi: 10.1186/s12885-022-09269-x (PMC8832637; doi:10.1186/s12885-022-09269-x)

Supplementary table 1 - Full list of references and acronyms for the PROMs

| **Acronym** | **Tool** | **References** |
| --- | --- | --- |
| Breast-Q | Breast Questionnaire | Pusic AL, Klassen AF, Scott AM, Klok JA, Cordeiro PG, Cano SJ. Development of a new patient-reported outcome measure for breast surgery: the BREAST-Q. Plast Reconstr Surg 2009 Aug;124(2):345-353. |
| DT | Distress Thermometer | National Comprehensive Cancer Network. Distress management. Clinical practice guidelines. J Natl Compr Canc Netw 2003 Jul;1(3):344-374. |
| DN4 | Douleur Neuropathique 4 | Bouhassira D, Attal N, Alchaar H, Boureau F, Brochet B, Bruxelle J, et al. Comparison of pain syndromes associated with nervous or somatic lesions and development of a new neuropathic pain diagnostic questionnaire (DN4). Pain 2005;114(1-2):29-36. |
| EORTC PATSAT | European Organization for Research and Treatment of Cancer – Patient Satisfaction Questionnaire | Bredart A, Bottomley A, Blazeby J, Conroy T, Coens C, D’Haese S, et al. An international prospective study of the EORTC cancer in-patient satisfaction with care measure (EORTC IN-PATSAT32). Eur J Cancer 2005;41(14):2120-2131. |
| EORTC QLQ-C30 | European Organization for Research and Treatment of Cancer – Quality of Life Core Questionnaire | Aaronson NK, Ahmedzai S, Bergman B, Bullinger M, Cull A, Duez NJ, et al. The European Organization for Research and Treatment of Cancer QLQ-C30: a quality-of-life instrument for use in international clinical trials in oncology. JNCI: Journal of the National Cancer Institute 1993;85(5):365-376. |
| EQ-5D | EuroQol Group – 5-Dimension questionnaire | Longworth L, Rowen D. Mapping to Obtain EQ-5D Utility Values for Use in NICE Health Technology Assessments. Value in Health 2013 January–February 2013;16(1):202-210. |
| ESAS | Edmonton Symptom Assessment Scale | Bruera E, Kuehn N, Miller MJ, Selmser P, Macmillan K. The Edmonton Symptom Assessment System (ESAS): a simple method for the assessment of palliative care patients. J Palliat Care 1991;7(2):6-9. |
| FACT-G | Functional Assessment of Cancer Therapy – General module | Weitzner MA, Meyers CA, Gelke CK, Byrne KS, Levin VA, Cella DF. The Functional Assessment of Cancer Therapy (FACT) scale. Development of a brain subscale and revalidation of the general version (FACT‐G) in patients with primary brain tumors. Cancer 1995;75(5):1151-1161. |
| HADS | Hospital Anxiety and Depression Scale | Zigmond AS, Snaith RP. The Hospital Anxiety and Depression Scale. Acta Psychiatr Scand 1983 06/01; 2021/02;67(6):361-370. |
| IPSS | International Prostate Symptom Score | Barry MJ, Fowler Jr FJ, O’Leary MP, Bruskewitz RC, Holtgrewe HL, Mebust WK, et al. Correlation of the American Urological Association symptom index with self-administered versions of the Madsen-Iversen, Boyarsky and Maine Medical Assessment Program symptom indexes. J Urol 1992;148(5):1558-1563. |
| MAC/mini-MAC | Mental Adjustment to Cancer – Regular and short version | Watson M, Greer S, Young J, Inayat Q, Burgess C,  Robertson B. 1988. Development of a questionnaire  measure of adjustment to cancer: The MAC scale.  Psychol Med 18: 203–209  Watson M, Greer S, Young J, Inayat Q, Burgess C,  Robertson B. 1988. Development of a questionnaire  measure of adjustment to cancer: The MAC scale.  Psychol Med 18: 203–209  Watson M, Greer S, Young J, Inayat Q, Burgess C,  Robertson B. 1988. Development of a questionnaire  measure of adjustment to cancer: The MAC scale.  Psychol Med 18: 203–209  Watson M, Greer S, Young J, Inayat Q, Burgess C, Robertson B. Development of a questionnaire measure of adjustment to cancer: the MAC scale. Psychol Med 1988;18(1):203-209. |
| MMPI | Minnesota Multiphasic Personality Inventory | Hathaway SR, McKinley JC. A multiphasic personality schedule (Minnesota): I. Construction of the schedule. J Psychol 1940;10(2):249-254. |
| Morse S | Morse Scale | Morse JM, Morse RM, Tylko SJ. Development of a Scale to Identify the Fall-Prone Patient. Canadian Journal on Aging / La Revue canadienne du vieillissement 1989;8(4):366-377. |
| MOS SF-36/SF-12 | Medical Outcome Survey Short Form – 36- and 12-item versions | Ware Jr JE, Sherbourne CD. The MOS 36-item short-form health survey (SF-36): I. Conceptual framework and item selection. Med Care 1992:473-483. |
| NEQ | Needs Evaluation Questionnaire | Tamburini M, Gangeri L, Brunelli C, Beltrami E, Boeri P, Borreani C, et al. Assessment of hospitalised cancer patients' needs by the Needs Evaluation Questionnaire. Annals of Oncology 2000;11(1):31-38. |
| PDI | Psychological Distress Inventory | Morasso G, Costantini M, Baracco G, Borreani C, Capelli M. Assessing psychological distress in cancer patients: validation of a self-administered questionnaire. Oncology 1996 Jul-Aug;53(4):295-302. |
| PedsQL | Pediatric Quality of Life Inventory | Varni JW, Seid M, Rode CA. The PedsQL™: measurement model for the pediatric quality of life inventory. Med Care 1999:126-139. |
| PRO-CTCAE | Patient-Reported Outcome- Common Terminology Criteria for Adverse Events | Basch E, Reeve BB, Mitchell SA, Clauser SB, Minasian LM, Dueck AC, et al. Development of the National Cancer Institute’s patient-reported outcomes version of the common terminology criteria for adverse events (PRO-CTCAE). J Natl Cancer Inst 2014;106(9):dju244. |
| PROMIS | Patient-Reported Outcomes Measurement Information System | Ader DN. Developing the patient-reported outcomes measurement information system (PROMIS) 2007. |
| STAI/SDS | State-Trait Anxiety Inventory | Spielberger CD. State-trait anxiety inventory: a comprehensive bibliography: Consulting Psychologists Press; 1989. |

Supplementary figure 1 – Percentage distributions regarding who should propose PROMs to patients and who should help patients fill them out.


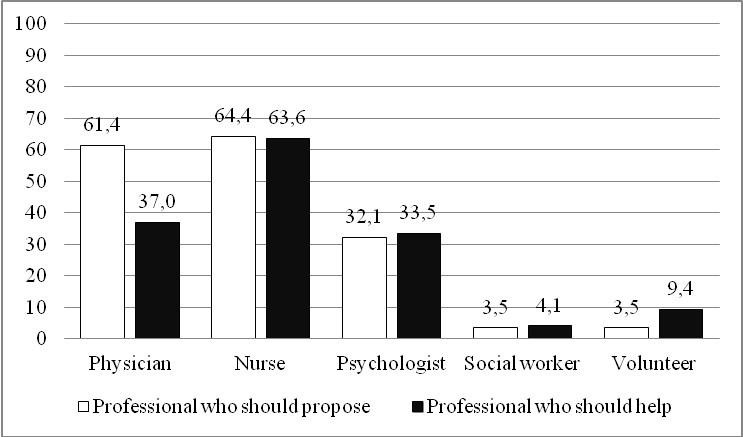

Supplement: Supplementary file 1 — Additional file 1. [file 12885_2022_9269_MOESM1_ESM.docx]
